# Interplay Between Endolysosomal Calcium Signalling, EMT, and Autophagy in Colorectal Cancer (CRC): Implications for Metastasis and Therapeutic Strategies

**DOI:** 10.34172/apb.45688

**Published:** 2026-06-27

**Authors:** Nur Aqilah Nadhirah Affendie, Nurul Syahmin Ab Aziz, Siti Yusrina Nadihah Jamaludin, Normala Abd Latip, Aisyah Hasyila Jahidin

**Affiliations:** ^1^Faculty of Pharmacy, Universiti Teknologi MARA (UiTM), Cawangan Selangor, Puncak Alam Campus, Selangor, Malaysia; ^2^School of Life Sciences, University of Warwick, Coventry, CV4 7AL, UK; ^3^Faculty of Medicine, Universiti Sultan Zainal Abidin, Medical Campus, Terengganu, Malaysia

**Keywords:** Autophagy, Calcium signalling, Colorectal cancer, Epithelial-mesenchymal transition, EMT, Metastasis, TRPML1

## Abstract

Colorectal cancer (CRC) is a form of cancer that, while initially slow to develop, becomes aggressive and poses significant treatment challenges in its later stages, often resulting in a low survival rate. This progression is evident, with patients having an approximate 90% five-year survival rate at the localised stage CRC, which drops dramatically to 12.5% for those with distant metastases. Epithelial-to-mesenchymal transition (EMT) plays a pivotal role in metastases, as it facilitates the spread of cancer cells to migrate from the primary site to secondary locations. Autophagy further supports this process by enhancing cell survival under stress. Both EMT and autophagy are tightly regulated by calcium signalling, which is essential for cancer cell migration, invasion, and survival under unfavourable conditions. Targeting specific calcium channels and signalling pathways can inhibit EMT and regulate autophagy, thereby reducing cancer metastasis. This review explores the intricate relationship between endolysosomal (EL) calcium signalling, EMT, and autophagy in CRC, highlighting potential therapeutic strategies and the significance of these findings for improving treatment outcomes.

## Introduction

 Colorectal cancer (CRC) is a major global health concern, ranking third in incidence and second in mortality worldwide, according to the World Health Organisation (WHO).^1^ Projections indicate a significant increase in CRC cases and deaths by 2040.^2^ CRC frequently originates from benign polyps in the colon or rectum,^3^ which can develop into cancerous growths over time. Adenocarcinoma represents the predominant histological type,^4^ comprising over 90% of CRC cases globally.^5^

 Despite improvements in diagnosis and treatment, CRC can become metastatic,^6^ posing significant challenges and resulting in low overall survival (OS) rates. The five-year survival rate for localised CRC is approximately 90%,^7^ but it decreases to 70.4% for regional involvement and dramatically drops to 12.5% for distant metastases.^7–9^ Metastasis occurs when cancer cells invade surrounding tissue, enter blood or lymphatic vessels, and establish secondary tumours in other organs.^10,11^

 Cellular mobility is fundamental to driving metastasis.^12^ Cells may undergo epithelial-to-mesenchymal transition (EMT), involving significant alterations in epithelial cells to adopt a mesenchymal phenotype. This change is defined by decreased cell polarity and adhesion and increased migratory and invasive abilities. EMT plays a crucial role in cancer metastasis, facilitating the spread of cancer cells from the original location to secondary sites.^6^ The acquisition of mesenchymal features increases cancer cells’ capacity to detach from the primary tumour, penetrate the extracellular matrix, and spread through the bloodstream, significantly impacting cancer severity and mortality rates.

 Autophagy has been implicated in metastatic progression.^13^ It is an intracellular process involving the degradation and recycling of cellular components to reduce stress and enable survival under challenging circumstances such as oxidative stress, hypoxia, and nutrient deprivation.^14^ In cancer metastasis, autophagy can function as both a suppressor and promoter, depending on the stage of cancer progression.^13^ It is essential for the survival of EMT cells during the process of migration and dissemination, and regulates the production of EMT markers in some cancer types.^15^ Autophagy’s role in cancer formation varies based on factors such as nutrition availability, microenvironment stress, pathogenic circumstances, acting neutrally, suppressively or promotively towards tumour growth.^16^

 Calcium signalling modulates both the EMT and autophagy processes. The interplay between EL calcium signalling, EMT and autophagy outlines the complexity of cellular regulation in CRC. Modulating EL calcium can drive the dynamic changes required for metastasis and the metabolic adaptability of cancer cells through autophagy. This opens possibilities for targeting specific channels and signalling pathways to prevent EMT, modulate autophagy, and develop effective therapeutic strategies to combat cancer onset, metastasis and resistance to conventional therapies. This review systematically explores six key areas: the epidemiology of CRC, the interplay between EMT and cancer metastasis in CRC, the role of autophagy in cancer progression and CRC, the involvement of calcium signalling in CRC, the interlink between calcium signalling, EMT, and autophagy, and therapeutic targets and strategies in CRC treatment.

## Epidemiology of Colorectal Cancer (CRC)

 The GLOBOCAN 2020 data by the WHO reveals that there were almost 19.3 million new CRC cases and nearly 10 million CRC-related deaths worldwide. CRC incidence varies geographically, with the highest rates in North America and Europe, while regions like Algeria have lower but increasing rates, growing at approximately 7% annually.^17^ In Algeria, CRC is the second most prevalent cancer after breast cancer, with standardised rates of 23 per 100,000 in males and 16 per 100,000 in females.^17^ Rectal cancer accounts for 49.66% of cases, and colon cancer for 49.09%.^18^ The sigmoid colon is the most frequently affected site, followed by the ascending, transverse, descending colon, cecum, and crossing site.^18^ The GLOBOCAN 2020 estimates for Southeast Asia indicate that Indonesia will see the highest increase in new cancer cases, from 35,676 in 2022 to 61,109 in 2045, a 71.3% rise (Figure 1).^19^ The number of deaths in Indonesia is expected to increase from 19,255 in 2022 to 35,825 in 2045, a 86.1% rise (Figure 2).^19^ The Philippines, Thailand, and Malaysia will also experience substantial increases in both new cases and cancer-related deaths (Figure 2).^19^

**Figure 1:**
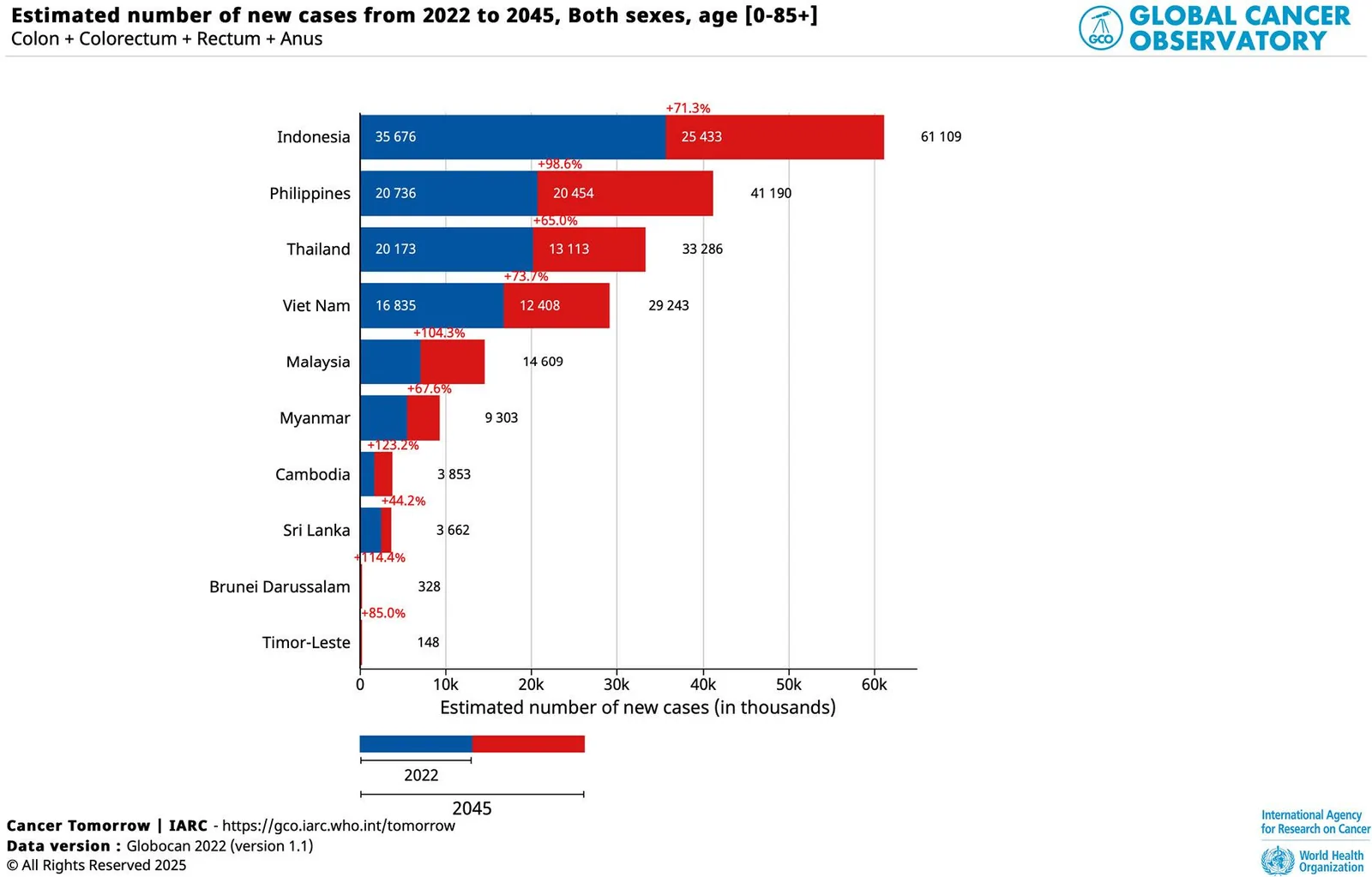
The estimated number of new cases from 2022 to 2030 for both sexes (reproduced from https://gco.iarc.fr)^19^

**Figure 2:**
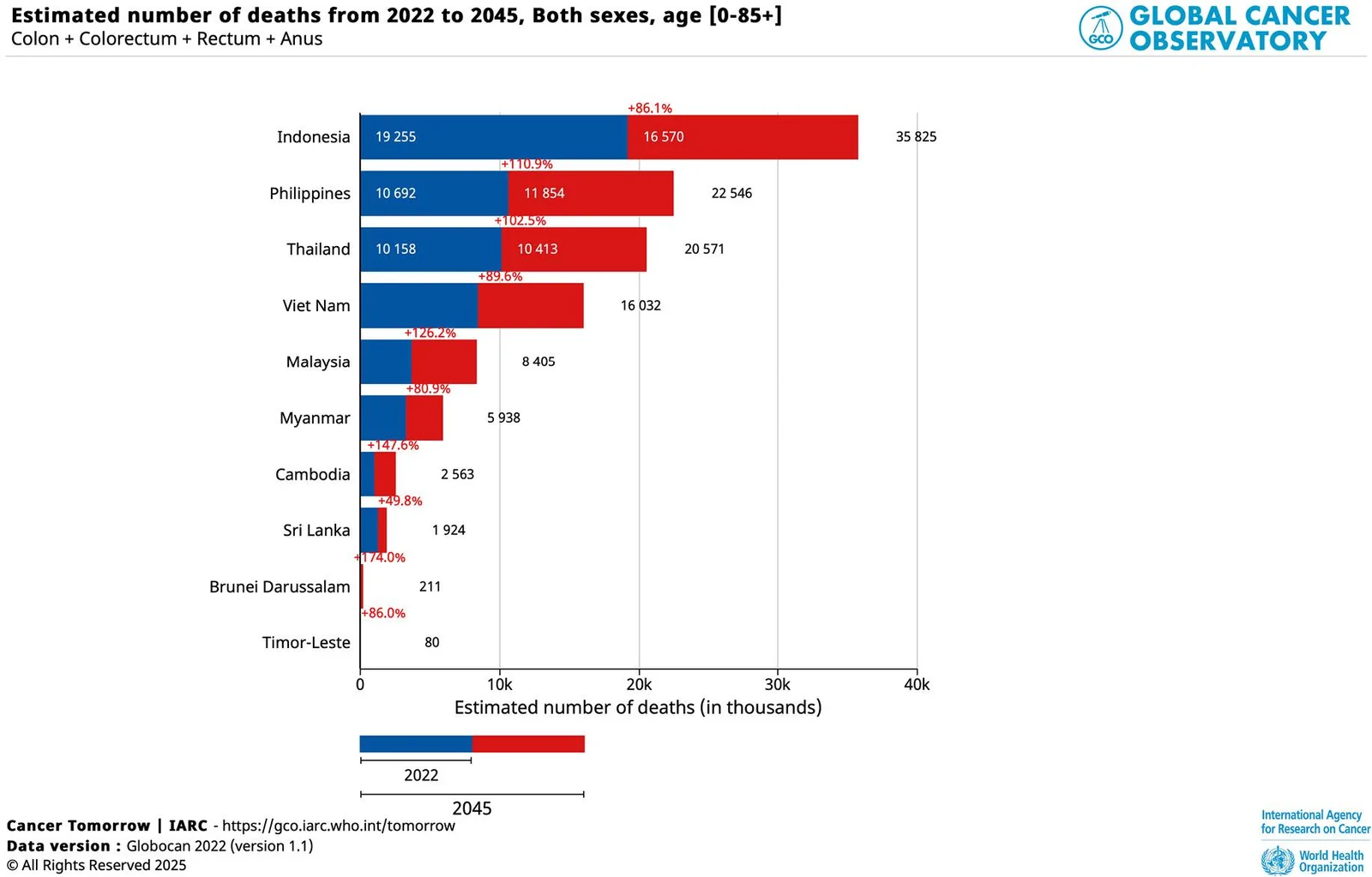
The estimated number of deaths from 2022 to 2030 for both sexes (reproduced from https://gco.iarc.fr)^19^

 In Malaysia, CRC is the second most prevalent form of cancer, accounting for 14.1% of new cases between 2017 and 2021.^20^ The National Strategic Plan for Colorectal Cancer (NSPCRC) 2021-2025 states that CRC is the most common cancer in men and the second most common in women, with a higher prevalence among the Chinese population compared to Malays and Indians.^21^ Despite being preventable and treatable through early screening, over 70% of CRC cases in Malaysia are diagnosed at advanced stages, contributing to a lower 5-year survival rate compared to other developed Asian countries.^22^

## Calcium Signalling in CRC

 Intracellular calcium ion (Ca^2+^) is a ubiquitous second messenger that regulates various signalling pathways within cells. The influx and efflux of Ca^2+^ are mediated by calcium channels, pumps, and exchanger networks,^23^ found in both internal and external cell membranes.^24^ Commonly studied Ca^2+^ channels for cancers include voltage-gated calcium channels (VGCCs), store-operated channels (SOCs), and transient receptor potential (TRP) channels.

 VGCCs, located on the cell membrane, open in response to changes in electrical membrane potential, allowing Ca^2+^ entry. These channels are encoded by ten genes, forming three subfamilies: Ca_v_1, Ca_v_2, and Ca_v_3, each with unique electrophysiological characteristics.^23,25^ Notably, the Ca_v_1.3 subtype is significantly upregulated in HCT116 cells, associated with increased basal cytosolic calcium levels and enhanced cell migration, crucial for metastasis.^22,24^

 Calcium influx triggered by the depletion of endoplasmic reticulum (ER) calcium stores is primarily mediated through a mechanism known as store-operated calcium entry (SOCE), which relies on SOCs.^26,27^ In this pathway, stromal interaction molecule (STIM) proteins act as calcium sensors within the ER. Upon store depletion of Ca^2+^, STIM proteins translocate to the ER-plasma membrane junctions to interact with ORAI1 proteins, forming calcium release-activated calcium (CRAC) channels.^26,27^ These channels exhibit high calcium selectivity and are essential for maintaining calcium homeostasis.^26,28^ In CRC, overexpression of STIM1, STIM2, and particularly ORAI1 enhances calcium influx, promotes proliferation, migration, and EMT.^29,30^

 TRP channels are non-selective cation channels that allow the passage of both monovalent and divalent cations,^24^ with a preference for K+, Na+, and Ca^2+^. They play vital roles in various disorders, such as metabolic, cardiovascular, and cancer.^31,32^ High expression of transient receptor potential ankyrin 1 (TRPA1) in metastatic CRC cells leads to mitochondrial dysfunction and apoptosis (Table 1) due to excessive Ca^2+^ entry, triggered by reactive oxygen-species (ROS) and activation of caspase-3/7.^33^

**Table 1:** Key calcium channels in CRC

**Ca**^2+^ **channels**		**Expression**		**Reference**
		**Gene**	**Protein**	
VGCCs	Ca_v_1.2	↑		^24,34,35^
	Ca_v_1.3	↑	↑	^24,34^
	Ca_v _3.1	↑		^24,34^
TRP	TRPA1	↑		
	TRPC1	↑	↑	^36^
	TRPM6	↓		^24,37^
	TRPM7	↑	↑	^38,39^
	TRPM8	↑	↑	^40^
	TRPV6	↑		^41,42^
	TRPML1	↓		^43^
	TRPML2	↓		^43,44^
SOC	ORAI1	↑	↑	^29^
	STIM1	↑	↑	^45^
	STIM2	↑	↓	^24,46^

Expression profiles of key calcium channels implicated in CRC. The table includes voltage-gated calcium channels (VGCCs), transient receptor potential (TRP) channels, and store-operated calcium (SOC) channels. Expression levels are categorised at the gene and protein levels. ↑ indicated increased expression; ↓ indicates decreased expression

 Endolysosomal (EL) calcium channels, notably the two-pore channels (TPCs) and TRP mucolipin channels (TRPMLs), have emerged as key intracellular regulators of calcium signalling in cancer, including CRC. Embedded within the EL system, these channels mediate Ca^2+^ release from acidic organelles such as lysosomes and endosomes,^47^ impacting vesicular trafficking, autophagy, and mTOR signalling. Dysregulation of their activity contributes to disrupted Ca^2+^ homeostasis and supports processes such as tumourigenesis, metastasis, and therapy resistance.^48^ TPC2, for instance, has been shown to enhance cancer cell migration and invasiveness.^49^ At the same time, TPC1 mediates the nicotinic acid adenine dinucleotide phosphate (NAADP)-induce calcium release, thus activating the ERK and PI3K/AKT pathways, promoting proliferation in metastatic CRC cells.^47,48^ TRPML1 influences intracellular trafficking of adhesion molecules such as E-cadherin and β1-integrin, thereby modulating motility.^50^

 Calcium signalling is critical in CRC cell migration, invasion, and survival by regulating processes like actin cytoskeleton dynamics, EMT, and cellular proliferation. It orchestrates actin-dependent migration and invasion through calcium-regulated molecules like calmodulin and calcium-activated protein kinases, enhancing metastatic potential.^51^ Furthermore, calcium signalling facilitates EMT, increasing cell motility and invasiveness.^52^ While slight upregulation promotes proliferation, excessive calcium influx can induce cell death, reflecting its dual role in tumour dynamics.^53^ Clinically, hypercalcemia in CRC patients is linked to increased tumour growth and metastasis.^53^

 Specific calcium channels and pathways are key mediators in CRC progression. TRPM4, downregulated in CRC due to promoter methylation, inhibits tumour growth and metastasis when restored, activating the Ca^2+^/calpain pathway, leading to focal adhesion kinase (FAK) proteolysis and suppression of the PI3K/Akt/mTOR pathway.^54^ TRPC1, upregulated in CRC, promotes aggressive tumour behaviour by activating the PI3K/AKT signalling axis through interaction with calmodulin (CaM). Knockdown of TRPC1 reduces cell proliferation and tumour growth.^36^ Higher calcium release-activated calcium modulator 1 (ORAI1) expression is associated with increased metastasis, and its knockdown suppresses EMT and migration (Figure 3).^29^ Other channels, such as KCNN4, TRPM2, and ORAI3, also influence CRC progression, with altered expression patterns influencing prognosis. For instance, an increased ORAI3/ORAI1 ratio is associated with advanced CRC and poorer outcomes.^55^

**Figure 3:**
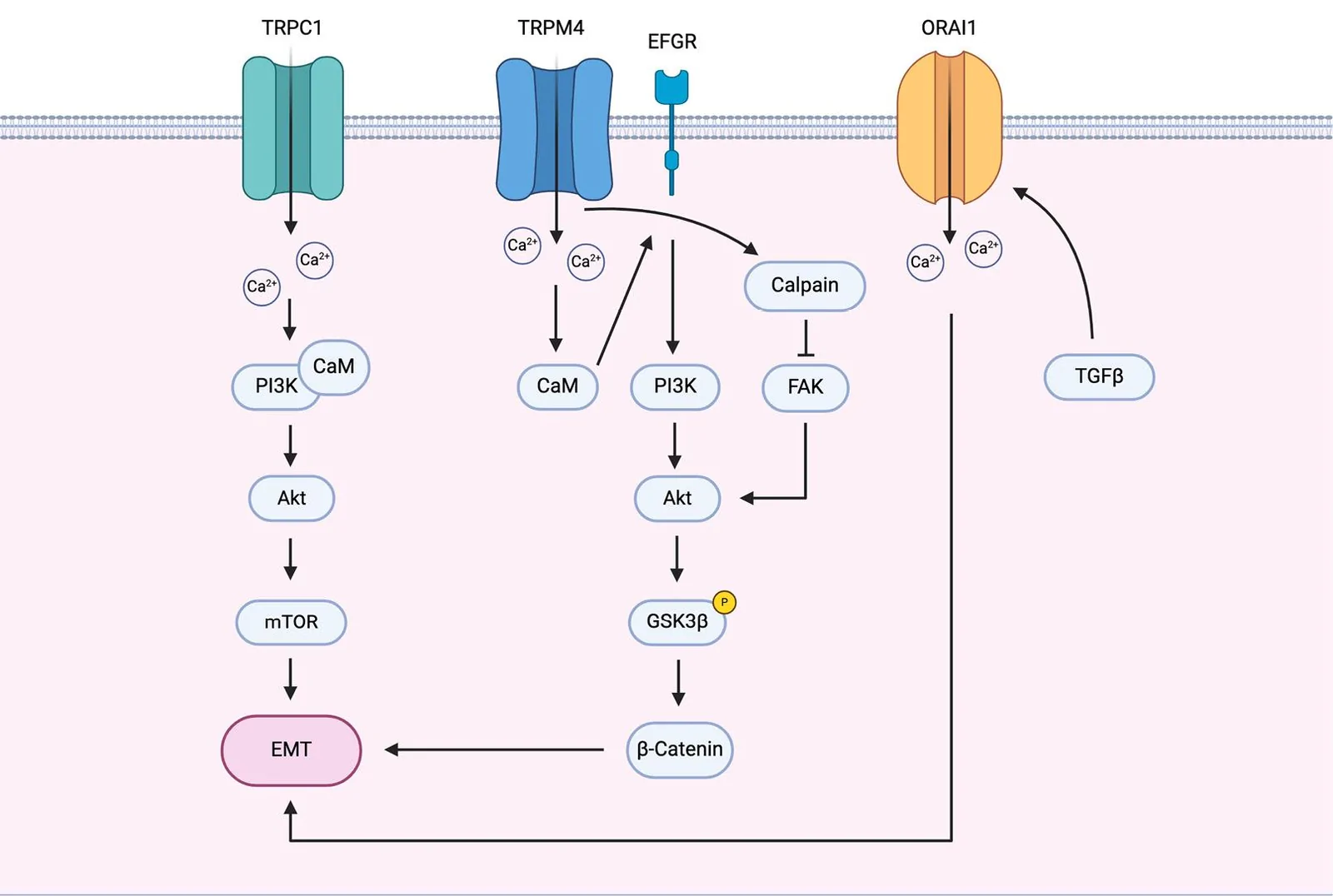
Key calcium channels and signalling pathways involved in CRC

## Interplay Between EMT and Cancer Metastasis

 EMT enhances cancer cell migration and colonisation at distant sites. Epithelial cells, typically stationary and forming tight layers, gain motility and invasive properties, transforming into mesenchymal cells.^56^ This transition is vital in embryonic development, wound healing, and cancer metastasis.^57,58^

 EMT-driven cancer invasion involves complex signalling networks and transcription factors. A hallmark of EMT is the downregulation of epithelial marker expression (e.g. E-cadherin) and upregulation of mesenchymal markers (e.g. vimentin), enhancing cancer cell invasiveness.^59^ Aberrant expression of markers like E-cadherin, Vim, β-catenin, and SMAD4 correlates with poor outcomes, including decreased overall survival (OS) and disease-free survival (DFS), and higher metastasis rates.^60^

 Advanced techniques like single-cell RNA sequencing and spatial transcriptomics have improved better understanding of EMT’s role in revealing that EMT not only increases malignant epithelial cells but also induces their differentiation into cancer-associated fibroblasts (CAFs). These CAFs drive tumour invasion and metastasis through activation of pathways such as TGF-β signalling and angiogenesis.^61^ In prostate cancer, markers like cytokeratin 8 (CK8) and Vim are linked to higher Gleason scores and biochemical recurrence, indicating their potential in predicting disease progression and prostate-specific antigen (PSA) treatment failure.^62^

 EMT markers such as E-cadherin, N-cadherin, SLUG, SNAI1, and TWIST in pituitary adenomas correlate with tumour size, staging, and endocrine functions, making them useful for assessing tumour aggressiveness and predicting outcomes.^63^ SLUG is a key regulator of EMT in various cancers, including breast, colon, ovarian, and head and neck squamous cell carcinomas, with high expression in both in vitro and in vivo settings.^64^ New markers like phospholipid phosphatase 4 (PLPP4) are being identified to deepen understanding of EMT and its impact on cancer progression and treatment resistance.^65^

 Tumour budding, a result of EMT, is linked to unfavourable outcomes in early-stage ovarian clear cell carcinoma, with abnormal E-cadherin and β-catenin expression in tumour buds suggesting Wnt signalling involvement.^66^ MALAT1, a long non-coding RNA, is crucial in EMT regulation, influencing marker expression and promoting cancer cell spread.^67^

 EMT regulation involves signalling pathways like TGF-β, Wnt/β-catenin, Notch, Sonic Hedgehog (SHH) and receptor tyrosine kinases, which activate EMT. EMT transcription factors (EMT-TFs) include SNAI1, TWIST, and ZEB1/2.^64,68,69^ These pathways are interconnected, with crosstalk and feedback loops.^69,70^ EMT is induced by factors like hypoxia, inflammatory cytokines (NF-κB/TNF-α/IL6), and oncogenic signals, leading to epithelial marker downregulation and mesenchymal marker upregulation.^58,68^

 Metabolic remodelling in EMT involves glycolysis, the Kreb’s cycle, and fatty and amino acid metabolism, with genetic and transcriptional changes enhancing invasive and metastatic phenotypes.^57^ EMT is associated with cell death like apoptosis, ferroptosis, net-autophagy (NET) and necroptosis, influencing immune evasion and metastasis.^71^

 Metastasis-specific EMT (msEMT) genes in gastric cancer show differential expression based on metastasis mode, indicating unique biological pathways for each route.^72^ The Tri-PyMT EMT lineage tracing model in breast cancer shows pre-EMT cells are primarily responsible for metastasis, whereas post-EMT cells drive tumour invasion and angiogenesis, highlighting EMT’s evolutionary and flexible role in cancer progression.^73^

## EMT Interplay in Colorectal Cancer (CRC)

 Common EMT-TFs expressed at post-transcriptional and post-translational levels in CRC include TWIST1, SNAIL, SLUG, and ZEB1/2.^59^ These genes suppress epithelial markers to promote a mesenchymal phenotype, which is essential for metastasis. Furthermore, several microRNAs (miRNAs) have been identified as regulators of EMT. For example, miR-17 overexpression leads to the degradation of cytochrome P450, family 7, subfamily B, polypeptide 1 (CYP7B1) mRNA, which is associated with elevated EMT in DLD1 cells.^74^ Additionally, miR-17-5p upregulation in HT29 and LoVo cells decreases Vim expression, thereby inhibiting cell migration and invasion.^75^ Moreover, Sun et al^76^ reveals that miR-335-5p facilitates EMT by targeting RAS p21 protein activator 1 (RASA1), while overexpression of RASA1 negates this effect in SW620 cells.

 The transcription factor BHLHE40 has been discovered as a crucial controller of EMT, significantly stimulating cell growth, invasion, migration, and liver metastasis in CRC.^61^ The clinical importance of EMT markers such as Snail and E-cadherin has been emphasised in aggressive CRC phenotypes. Snail facilitates invasion and metastasis by reducing E-cadherin expression, making both Snail and E-cadherin potential prognostic markers for CRC.^77^ Additionally, the EMT process is influenced by various signalling pathways like TGF-β, which can initiate EMT and regulate cancer progression, its microenvironment, and immunity resistance in CRC.^59^

 The PI3K/AKT signalling pathway is another key element in the EMT process. This pathway plays a crucial role in promoting the spread of CRC by reducing the expression of epithelial markers and increasing the expression of mesenchymal markers and particular transcription factors associated with EMT.^78^ Furthermore, the TGF-β signalling pathway is notably enhanced in metastatic primary tumours, playing a role in EMT and subsequent liver metastasis.^61^ Figure 4 illustrates the key pathways involved in EMT, including Wnt/β-catenin, PI3K/AKT, and TGF-β signalling, as well as their impact on transcription factors and gene expression relevant to CRC metastasis.

**Figure 4:**
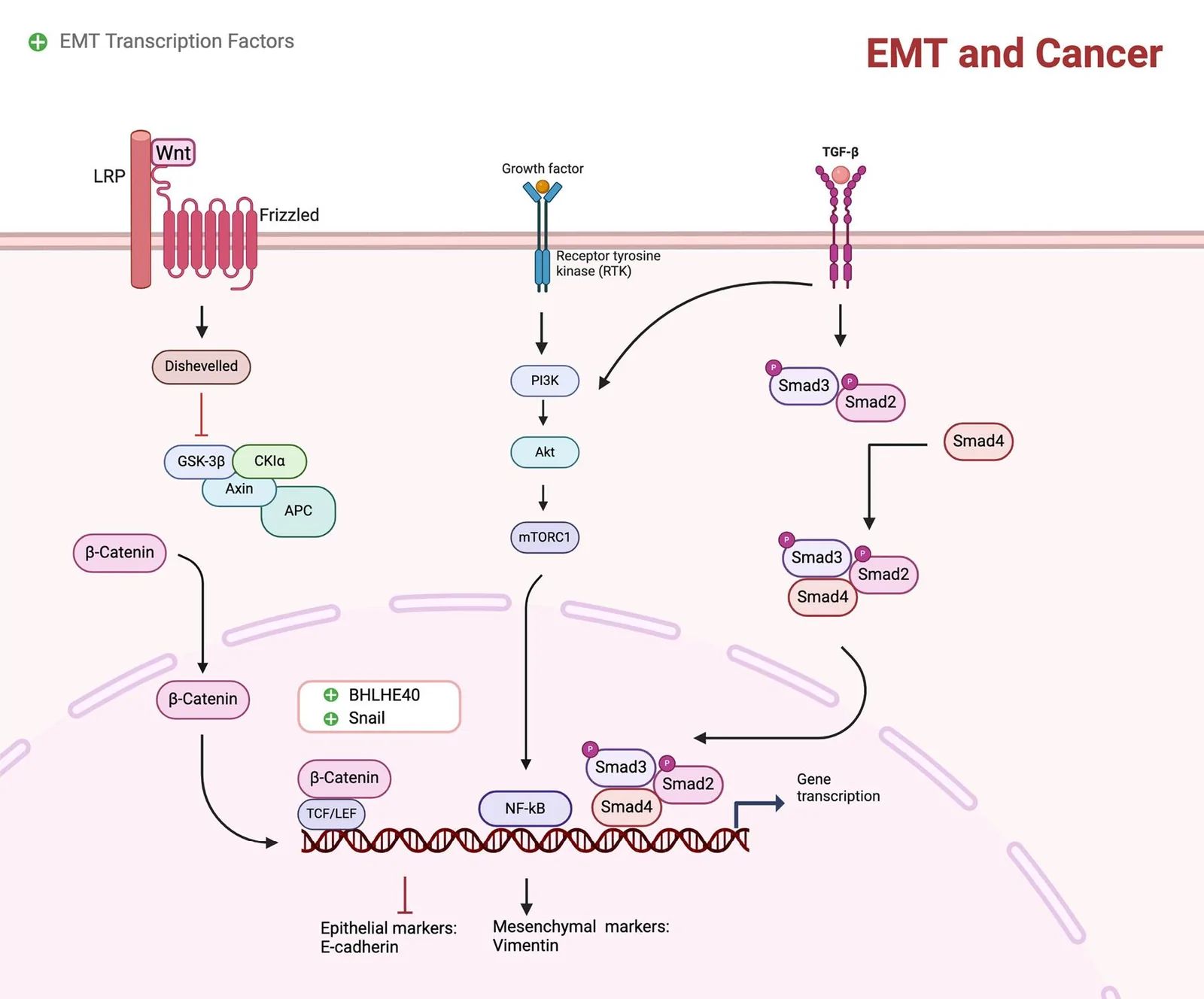
The key pathways involved in EMT

 Non-coding RNAs, such as long non-coding RNAs (lncRNAs) and circular RNAs (circRNAs), also play a crucial role in regulating EMT through competitive endogenous RNA (ceRNA) networks, interacting with microRNAs to control the expression of EMT-related genes.^79,80^ Proteomic studies have identified new EMT-related proteins, including GNG2, COL6A1, and CAVIN1, which are linked to CRC metastasis, particularly in the liver.^81^ Metabolic abnormalities, especially those related to the tricarboxylic acid cycle and enzymes such as succinate dehydrogenase, are also involved in the activation of EMT, connecting metabolic changes to tumour growth.^82^

 The relevance of EMT in CRC metastasis is underscored by its dynamic and non-binary nature, governed by a complex network of intrinsic and extrinsic factors such as transcription factors, post-translational modifications, and epigenetic changes.^83^ In addition, certain genes, namely CXCL1, CXCL8, and MMPs, have been identified as modulators of EMT in CRC, highlighting the complex molecular landscape.^84^ The interaction between these pathways and factors not only influences the ability of CRC cells to spread to other parts of the body but also contributes to their ability to withstand chemotherapy and immunotherapy, which presents considerable difficulties in treatment.^80,83^ Understanding these pathways and their role in EMT and metastasis is essential for developing targeted treatments to improve patient prognosis.

 While EMT is a key driver of CRC metastasis, its regulation is further modulated by autophagy, a cellular process that maintains homeostasis but also plays a dual role in cancer progression. The interplay between EMT and autophagy is particularly crucial, as autophagy supports EMT cells during migration and contributes to therapy resistance. Understanding this dynamic interaction is vital for designing effective therapeutic interventions.

## Role of Autophagy in Cancer

 Autophagy plays a complex role in tumour progression, acting as both a promoter and suppressor depending on the cancer stage and circumstances.^85^ Initially, autophagy suppresses tumours by maintaining cellular balance and genomic integrity, thus preventing cancer onset.^86,87^ However, in advanced tumours, cancer cells exploit autophagy to meet metabolic demands and endure stress, promoting tumour growth and spread.^86–88^

 Autophagy regulates metastasis by controlling EMT.^15,89^ It stabilises transcription factors like Twist1, crucial for EMT and cancer cells dissemination. Blocking autophagy leads to p62/SQSTM1 accumulation, which binds to Twist1, preventing its breakdown and promoting EMT.^90^

 Furthermore, autophagy supports cancer stem cells (CSCs), essential for tumour recurrence, treatment resistance, and metastasis. It facilitates CSC survival and proliferation by modifying stress responses.^91^ The tumour microenvironment (TME), with factors like hypoxia and nutrient deprivation, also triggers autophagy, enhancing cancer cell survival and spread.^89^

 Autophagy’s dual role in metastasis is complex. It can enhance tumour cell survival and cancer spread but also inhibit extensive metastases by maintaining cellular dormancy and preventing excessive proliferation.^92^ Targeting autophagy in cancer treatment requires a careful and sophisticated strategy. Suppressing autophagy during early metastasis can hinder cancer spread, while enhancing it in later stages can prevent micro-metastases formation.^13,92^

## Role of Autophagy in CRC

 During the initial phases of CRC, autophagy functions as a tumour suppressor by maintaining DNA stability, promoting tumour cell death, and boosting immune surveillance, thereby hindering tumour onset and growth.^93^ However, as CRC progresses, autophagy can shift roles and facilitate tumour growth and spread by increasing tumour metabolism, mediating treatment resistance, and promoting EMT.^93–95^

 Abnormalities in the autophagy-related genes and ubiquitination processes in CRC tissues are linked to significant alterations in the transcriptional activity of genes such as PTEN-induced kinase 1 (PINK1) and protein tyrosine phosphatase non-receptor type 22 (PTPN22), suggesting their role in early cancer development and tumour progression.^96^ Sphingosine kinase 1 (SPHK1) is a key regulator of autophagy in CRC, promoting invasion and metastasis via the SPHK1-TRAF6-ULK1 signalling pathway.^97^

 The autophagy marker LC3 correlates with histological grade and TNM stage in CRC, with higher levels indicating a poor prognosis.^98,99^ Long non-coding RNAs (lncRNAs) also regulate autophagy, impacting CRC progression.^100^ Autophagy promotes EMT by breaking down cellular components and supplying the necessary energy and materials for the transition, aiding metastasis.^95^

 Overexpression of autophagy gene ATG16L1 is linked to a negative response to immunotherapy in microsatellite-stable CRC, suggesting autophagy’s role in immune evasion and tumour growth.^101^ Targeting autophagy could improve the effectiveness of chemotherapy, radiation, and immunotherapy. Researchers are exploring nanoparticles and autophagy modulators to manipulate autophagy for better cancer treatment outcomes.^94^

 Key proteins, including LC3, Beclin-1, ATG5, ATG4B, DAPK1, SERPINA1, LAMP-2, PINK1, and FOXO1, serve as important markers of autophagy in CRC. LC3 is crucial for autophagosomes formation and is linked to CRC prognosis.^99^ Beclin-1 overexpression is associated with poor survival outcomes in CRC patients undergoing chemotherapy.^101^ ATG5 and ATG4B are involved in autophagosome formation and play roles in CRC progression.^102^ DAPK1 and SERPINA1 are linked to CRC development, while LAMP-2 and PINK1 vary in expression across CRC stages, suggesting their diagnostic potential.^103^ FOXO1’s increase from early to advanced CRC stages highlights autophagy’s dynamic role in cancer.^103^

 Autophagy interacts with apoptosis, inflammation, and oxidative stress in CRC. It can either inhibit apoptosis, aiding tumour survival, or trigger cell death, impeding tumour growth.^93,96^ Hypoxia and oxidative stress in the TME modulate autophagy, promoting cancer spread.^104^ Autophagy-related gene expression is regulated by transcription factors, miRNAs, and RNA-binding proteins, maintaining autophagy levels during growth and stress.^105^

 Predictive models incorporating autophagy-related genes can forecast CRC prognosis and guide therapy.^106^ Targeting autophagy with nanoparticles and modulators shows potential in enhancing CRC treatment responsiveness.^93^ The dual role of autophagy in CRC highlights its therapeutic and prognostic potential, necessitating further research to fully understand its mechanisms and applications in cancer treatment.

 Along with others, the mitogen-activated protein kinase (MAPK) pathway regulates autophagy in CRC cells. demonstrated that cannabidiol (CBD) triggers autophagy in CRC cells via the MAPK pathway, involving JNK, p38, and ERK proteins.^107^ The mTOR signalling pathway also plays a role in regulating autophagy. A study by Zhu et al^108^ discovered that Qingjie Fuzheng granule (QFG), a traditional Chinese medicinal compound, stimulates autophagy in CRC cells by suppressing the mTOR pathway and enhancing the AMPK pathway. The PI3K/AKT pathway also plays a significant role in autophagy, as demonstrated by QFG’s effects on CRC cells.^109^

 Autophagy and apoptosis share common signalling pathways and protein components, with autophagy either inhibiting or promoting apoptosis depending on the cellular context and environmental factors.^108^ In addition, autophagy has a reciprocal relationship with ferroptosis, another mechanism of cell death, enhancing ferroptosis to inhibit cell growth and invasion.^110^

 Autophagy is crucial in chemoresistance, acting as a survival strategy during anticancer treatment. Suppressing autophagy can increase CRC cell sensitivity to chemotherapy, as evidenced by the impacts of 5-fluorouracil (5-FU) and oxaliplatin (OxaPt) on autophagy in CRC cell lines.^111^ Autophagy’s role in CRC is multifaceted, promoting and inhibiting tumour growth depending on the cancer progression stage.^112^

 Calcium is vital in regulating autophagy, influencing cancer cell survival and metastasis. In CRC, EL Ca^2+^ channels such as TRPML1, TPC2, and ORAI1 regulate autophagic flux, impacting therapy resistance and EMT-driven metastasis. Targeting calcium signalling to modulate autophagy offers a promising therapeutic strategy, but interventions must be tailored to the disease stage and context due to autophagy’s dual role in cancer progression.

## Interlink of Calcium Signalling in EMT and Autophagy

 Calcium signalling plays a crucial role in regulating both EMT and autophagy. The interplay between these processes involves multiple pathways and regulatory mechanisms. For instance, autophagy is regulated by pathways such as PI3K/AKT/mTOR, Beclin-1, p53, and JAK/STAT, which intersect with EMT-related pathways such as WNT, NF-kB, and TGF-β. Ca^2+^ acts as a second messenger, influencing cell proliferation, apoptosis, and autophagy, all interconnected with EMT. Specifically, calcium signalling modulates cell migration, invasion and angiogenesis by affecting the cytoskeleton and cell adhesion molecules, facilitating EMT.^51^

 In autophagy, calcium levels are crucial for forming pre-autophagosomal structures and omegasomes, which are the early steps in autophagosome formation.^113^ Interestingly, the inhibition of VGCCs activates autophagy in human adenocarcinoma, increasing apoptosis and reducing proliferation and migration. This suggests that tumour cells utilise Ca^2+^ to protect against autophagic death.^114^

 Recent studies have highlighted the role of EL TRPML1 in regulating migratory processes. Targeting TRPML1 could inhibit migration and invasion.^51^ TRPML1 is involved in ion balance, vesicle transport, and autophagy.^115,116^ It controls cancer cells migration, with its inhibition reducing breast cancer cell invasion *in vitro* and *in vivo*.^116,117^ Loss of TRPML1 disrupts E-cadherin and β1-integrin expression, reducing cell migration and adhesion.^42^ Despite the absence of CRC cells in the study, it is possible that comparable pathways involving TRPML1, which regulates cell motility and adhesion, may be involved in CRC. The disrupted trafficking of E-cadherin and β1-integrin resulting from the absence of TRPML1 activity may potentially result in decreased cell-cell adhesion and heightened metastatic capability in CRC.

 TRPML1 controls autophagy by transferring Ca^2+^, zinc (Zn^2+^), and iron (Fe^2+^) from lysosomes to the cytosol, which is crucial for the merging of autophagosomes with lysosomes.^118^ Activation of TRPML1 halts autophagy, leading to cell death in various cancers, such as pancreatic, breast, and stomach cancers. This occurs by interfering with the merging of autophagosomes and lysosomes during the fusion process.^119^ This interference is mediated by zinc, which hinders the connection between syntaxin 17 (STX17) in the autophagosome and VAMP8 in the lysosome.^120^ Moreover, the involvement of TRPML1 in autophagy is associated with its capacity to detect reactive oxygen species (ROS) and coordinate an autophagy-related process to alleviate oxidative stress, which is essential for cell survival during moderate stress but can induce autophagic cell death during severe stress conditions.^42^

 The interaction between TRPML1, autophagy, and EMT is apparent in the control of energy generation and protein synthesis during EMT. Autophagy sustains the required energy for EMT by preserving the balance of mitochondria, and hindering autophagy can impede EMT by diminishing ATP generation and suppressing protein synthesis.^120^ In pancreatic cancer, autophagy induced by CRT-mediated ER stress promotes EMT, increasing cell mobility and resistance to drugs.^121^ Blocking TRPML1 has been suggested as a potential treatment approach for cancer. Specific TRPML1 inhibitors, such as 17β-estradiol methyl ether (EDME) and similar compounds, have demonstrated effectiveness in suppressing the movement and invasion of triple-negative breast cancer cells by influencing autophagy and the movement of TFEB protein.^116^

 In CRC, autophagy promotes EMT, supporting tumour growth and chemotherapy resistance, thereby enhancing invasive and metastatic capabilities.^122^ The role of TRPML1 in autophagy is significant, as it hinders autophagosomes-lysosomes fusion, thereby halting the process of autophagic flux.^119^ Under high-stress conditions, this disturbance can result in autophagic cell death or apoptosis. This phenomenon could potentially be utilised to hinder the advancement of tumours in CRC.^42^ In addition, basal autophagy in CRC cells, which is not influenced by the mTORC1 pathway, controls the activation of receptor tyrosine kinases (RTKs) and the movement of cells. This further suggests that autophagy has a role in regulating the signalling and behaviour of cancer cells.^123^ Given TRPML1’s increased expression in various malignancies, including CRC, its regulation could modify autophagic responses and EMT, affecting tumour growth and metastasis.^43^ Hence, directing efforts towards TRPML1 to regulate autophagy offers a hopeful treatment approach in CRC, which could potentially impede EMT and decrease the spread of metastasis. To devise effective therapies, additional investigation is required to comprehensively understand the intricate relationships between TRPML1, autophagy, and EMT in CRC.^124^

## Therapeutic Targets and Strategies

 The regulation of EMT and autophagy involves intricate signalling networks, with calcium signalling playing a crucial role in these processes. Targeting specific components within these pathways offers promising therapeutic potential. For instance, store-operated calcium (SOC) channels, particularly ORAI1 and STIM1, mediate calcium entry into the cells. Inhibiting these channels disrupts calcium-dependent processes, enhancing differentiation, reducing autophagy, and potentiating anti-cancer agents like all-trans retinoic acid (ATRA).^125^ Similarly, calcium/calmodulin-dependent protein kinase kinase 2 (CAMKK2) links calcium signalling to autophagy. Its inhibition, along with its downstream effector AMP-activated protein kinase (AMPK), can suppress autophagy and EMT.^125^ Two-pore channel 2 (TPC2), a calcium channel within the EL system, is another key target, with inhibitors like naringenin and tetrandrine showing potential to modulate autophagy and EMT.^49^

 Current and emerging therapies for reducing CRC metastasis focus on targeting specific molecular pathways and leveraging advanced treatment strategies to address the challenges of metastatic disease, a leading cause of cancer-related mortality. Targeted molecular therapies, such as anti-vascular endothelial growth factor (VEGF) and anti-epidermal growth factor receptor (EGFR) agents, inhibit pathways critical for tumour angiogenesis and proliferation by blocking VEGF and EGFR, respectively.^126^ Precision medicine approaches targeting mutations like KRAS G12C, BRAF V600E, and HER2 overexpression offer promising personalised treatment options guided by advanced diagnostics.^127^ Emerging therapies include immune checkpoint inhibitors, which enhance the immune system’s ability to target cancer cells and are particularly effective in tumours with high microsatellite instability (MSI-H) or deficient mismatch repair (dMMR).^127^

 Neoadjuvant chemotherapy is increasingly used for initially resectable liver metastases, enabling tumour downsizing and assessment of biological behaviour, though patient selection is critical to mitigate potential hepatic injury.^128^ Innovative approaches, such as 3D tumour spheroid culture, improve drug screening by better mimicking the TME.^129^ Concurrently, nanodrug delivery systems aim to enhance the selectivity and efficacy of chemotherapeutic agents, despite challenges like drug-loading capacity and toxicity.^129^ Together, these advancements hold promise for improving therapeutic outcomes in metastatic CRC.

## Future Directions

 The interplay between EMT and autophagy in CRC highlights critical gaps in our understanding, particularly regarding their dual roles in tumour progression and treatment resistance. Autophagy exhibits a paradoxical function in CRC, acting as both a tumour suppressor and promoter, complicating its therapeutic targeting.^113,130^ Additionally, the relationship between autophagy and drug resistance remains poorly understood, especially regarding how its modulation could enhance chemosensitivity.^111^ Investigating EMT reversibility and its therapeutic modulation could lead to improved strategies for targeting metastatic CRC. Furthermore, the interaction between EMT and autophagy warrants further investigation, particularly their influence on cell adhesion and proliferation, as well as the reversibility of EMT – a factor critical for therapeutic interventions. Addressing these knowledge gaps is essential for advancing effective CRC treatment strategies.

## Conclusion

 The diagnosis of CRC remains a challenge due to its highly malignant and metastatic nature. Despite advancements in disease diagnosis and treatments, survival rates drop dramatically for patients with distant metastasis. EMT plays a crucial role in the metastatic progression of cancer, aiding cancer cells in migrating and invading other tissues. This process is regulated by Ca^2+^ signalling, which also influences autophagy. Inhibiting EMT, along with regulating autophagy and modulating Ca^2+^ signalling, is postulated to slow down metastatic progression and overcome resistance to conventional treatments. By focusing on these three events and gaining a more profound understanding of the molecular mechanisms governing CRC, future research can pave the way for more effective treatments, thus improving patient survival.

## Competing Interests

 The authors declare no conflict of interest.

## Ethical Approval

 Not Applicable.
